# Editorial: The significance of induced pluripotent stem cells in translational medicine

**DOI:** 10.3389/fimmu.2026.1965769

**Published:** 2026-08-26

**Authors:** Belen Alvarez-Palomo, Julia Skokowa, Helen Latsoudis

**Affiliations:** 1 Banc de Sang i Teixits, Barcelona, Spain; 2 Transfusional Medicine Group, Vall d’Hebron Institute for Research (VHIR), Barcelona, Spain; 3 Department of Oncology, Hematology, Clinical Immunology, and Rheumatology, University Hospital Tübingen, Tübingen, Germany; 4 Hemopoiesis Research Laboratory, School of Medicine, University of Crete, Heraklion, Greece; 5 Institute of Computer Sciences, Foundation for Research and Technology Hellas, Heraklion, Greece

**Keywords:** disease mechanisms, editorial, iPSCs, regeneration, translational medicine

## Introduction

Induced pluripotent stem cells (iPSCs) have revolutionized biomedical research by providing an ethically acceptable and virtually unlimited source of donor- and patient-specific pluripotent cells. Through the reprogramming of adult somatic cells, iPSCs can generate virtually any cell type, transforming disease modelling, drug discovery, regenerative medicine, and precision medicine by bridging basic research with clinical translation.

Over the past decade, patient-derived and isogenic iPSC models have deepened our understanding of disease mechanisms, enabled functional validation of pathogenic variants, and improved preclinical therapeutic testing. However, clinical translation remains limited by challenges including epigenetic memory, genomic instability, tumorigenicity, and variable differentiation efficiency.

This Research Topic brings together nine original research articles, reviews, and clinical perspectives that highlight recent advances in iPSC-based translational medicine.

## Disease modeling & cell replacement

In their research paper, De Homdedeu et al. developed a robust and scalable protocol for differentiating hiPSC lines into functional macrophages (iMacs), with immediate potential for good manufacturing practices (GMP)-compliant production. They optimized different culture conditions, including media composition, cytokine concentration, seeding density, culture format (2D vs. 3D) and substrate coatings across key developmental stages. Their protocol efficiently generated myeloid progenitors through embryoid bodies, producing highly pure iMacs (98% viable cells) from a single hiPSC, providing a promising platform for future cell-based immunotherapies.

The review by Mazurek et al. summarizes the current strategies for generating megakaryocytes and functional platelets from iPSCs *in vitro* and outlines the developmental foundations of primitive and definitive hematopoiesis that underpin these efforts. Furthermore, they describe strategies aimed at improving maturation and yield, along with emerging approaches in HLA editing and immune tolerance, which are designed to overcome alloimmune barriers in transfusion medicine and regenerative therapies.

The research paper by Alemany-Ribes et al. describes the generation of six iPSC lines as a model for investigating the molecular mechanisms underlying Multiple System Atrophy (MSA). Skin fibroblasts from patients with the two major MSA sub-types, MSA-P and MSA-C, with postmortem neuropathological confirmation in five cases, were reprogrammed into iPSC lines that fulfilled ISSCR quality standards and lacked acquired genetic abnormalities. Importantly, they differentiated into O4^+^ oligodendrocyte progenitor cells and MBP^+^ mature oligodendrocytes, providing a robust platform for studying MSA pathophysiology and therapeutic approaches.

Within the context of modeling myeloid cell development, Tesakov et al. review the expanding applications of iPSC technology in hemato-oncology, highlighting its major advantages, current limitations and future therapeutic potential: the use of scalable iPSC-derived blood cell therapies for bone marrow failure syndromes and leukemia, and CRISPR-Cas9 genome editing to model defective hematopoiesis, elucidate disease mechanisms, and facilitate drug discovery and repurposing. To this end, they review the role of *in silico* screening platforms, e.g. L1000CDS2 and the Connectivity Map, in prioritizing candidate therapeutic markers and supporting preclinical validation.

In their research article, Hutschalik et al. employed an iPSC-derived atrial tissue model to describe a novel inflammation-driven mechanism underlying atrial fibrillation (AF), the most common cardiac arrhythmia. Electrophysiological analyses demonstrated that M1 macrophage activation, combined with reduced expression of a series of AF associated markers, impaired electrical conduction and disrupted ion homeostasis. Computational tissue and whole-heart simulations confirmed that simultaneous reductions in sodium-potassium pump activity and tissue conductivity were sufficient to induce AF under tachycardic conditions even without ectopic activity, highlighting the value of combining iPSC models with *in silico* approaches to elucidate disease mechanisms and identify therapeutic targets.

## Challenges in iPSC applications

Despite significant advances, several challenges related to the risk of tumorigenicity due to genomic instability throughout reprogramming, long-term culture, lineage-specific differentiation and risk of residual undifferentiated cells are of concern for iPSC-based therapies.

To this end, Osete et al. present the key features of the Altered Genome Induced Immune Response hypothesis of iPSCs, which postulates that reprogramming-associated genomic and epigenetic alterations may induce cell-type specific immunogenicity in iPSC-derived products. Chromosomal insertions and deletions together with epigenetic modifications could alter differentiation programs and generate immunogenic cell populations. This hypothesis may explain some previously reported immune responses with iPSC-derived cells, such as T cell infiltration in teratomas or unexpected immunogenicity of autologous iPSC-derived cardiomyocytes (iPSC-CMs).

Within the same context, Kuebler et al. systematically evaluated the reprogramming efficiency of 150 iPSC lines by assessing the impact of donor characteristics, somatic cell source, culture conditions, reprogramming protocols, and passage numbers. Using diverse starting cell types, they minimized cell type-specific confounding effects. While technical parameters had only modest influence, donor-specific biological characteristics emerged as the primary determinants of reprogramming success, by affecting cell-cycle regulation, epigenetic remodeling, and responsiveness to reprogramming factors.

## Overcoming allogeneic immunogenicity through HLA-homozygous iPSC sources

The use of iPSCs lines from Human Leukocyte Antigen (HLA)-homozygous donors (i.e. haplo-lines) could be a mitigation strategy against the immunogenicity of iPSCs-derived transplants, especially during allogeneic regenerative cell therapies, since haplo-matching may profoundly expand the number of potential recipients.

In the original research, Maeding et al. showed that HLA-homozygous iPSC-CMs exhibit a stable immune phenotype, comparable to that of HLA-heterozygous cells, based on single-cell spectral flow cytometric analysis of 54 immune and pluripotency markers. Although HLA-homozygous iPSC-CMs presented lower HLA-class I expression, compared to their heterozygous counterparts, they retained a robust response to inflammatory stimulation, with interferon-γ up-regulating HLA-Class I, HLA-E, HLA-F, PD-L1, PD-L2, and CD47. These findings support the development of HLA-homozygous iPSC-CMs as semi-universal donor cells and establish spectral flow cytometry as a valuable platform for immune monitoring in cardiac cell replacement therapy.

Complementing this work, Naumovas et al. investigated the feasibility of establishing HLA-homozygous iPSC resources using gene editing on 3,496 high-resolution HLA typed donors from the Lithuanian Bone Marrow Donor Registry. 15 fibroblast lines were developed from triple HLA homozygotes for future iPSC derivation and immune compatibility studies. Finally, CRISPR-Cas9 guide RNA design yielded 54 candidates predicted to disrupt HLA-A or HLA-B while preserving HLA-C, producing edited profiles matching over 97.9% of Lithuanians, 95.7% of European American, and 95.5% of British populations.

**Figure 1:**
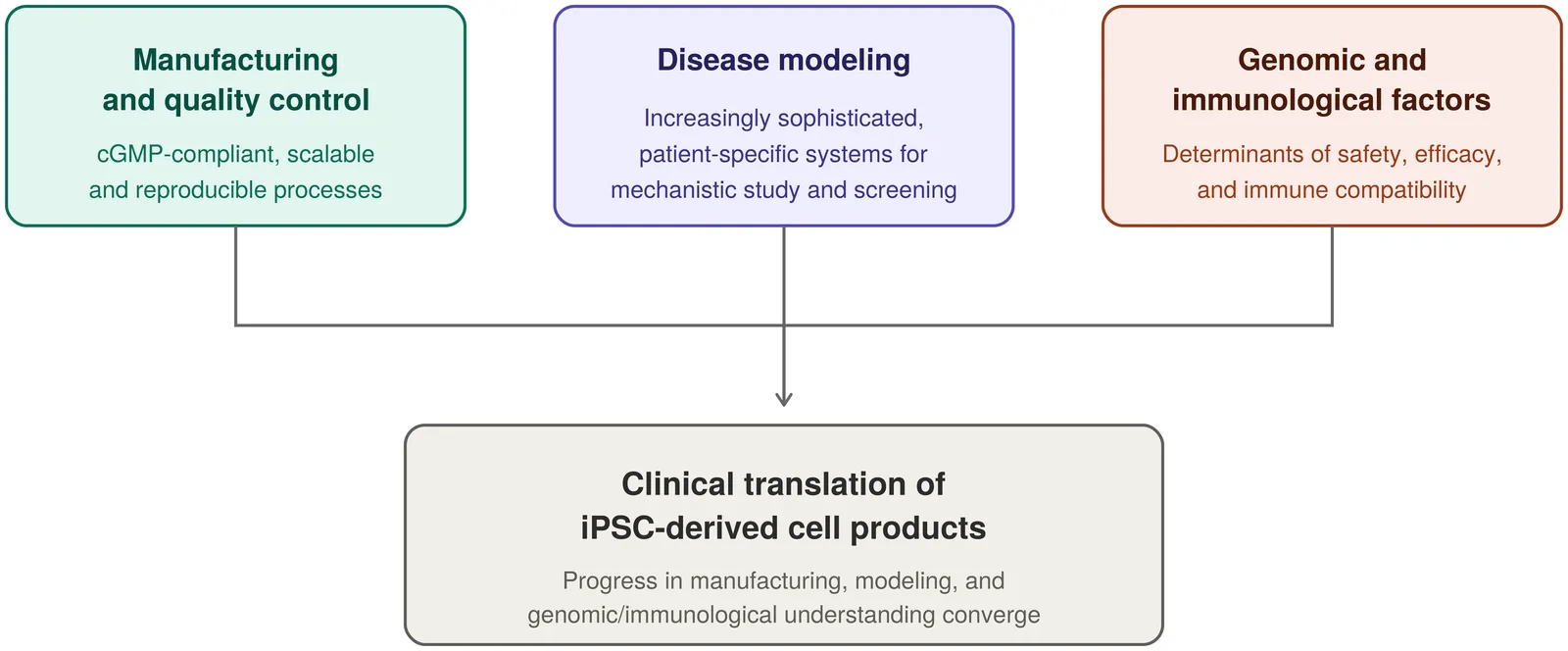
Three interconnected lines of work based on manufacturing and quality control, disease modelling and genomic/immunological characterization, may collectively advance the safe and effective clinical translation of iPSC-derived cell products.

In conclusion, this Research Topic highlights how recent advances in iPSC technology are accelerating the translation of iPSC-derived cell products toward clinical application (**Figure 1**).

